# Immunohistochemical and Histopathological Features of Persistent Gingival Enlargement in Relation to Metal Allergic Sensitisation during Orthodontic Treatment

**DOI:** 10.3390/ma16010081

**Published:** 2022-12-22

**Authors:** Martina Zigante, Stjepan Spalj, Jelena Prpic, Andrej Pavlic, Visnja Katic, Koviljka Matusan Ilijas

**Affiliations:** 1 Department of Orthodontics, Faculty of Dental Medicine, University of Rijeka, 51000 Rijeka, Croatia; 2 Clinical Hospital Center Rijeka, 51000 Rijeka, Croatia; 3 Department of Dental Medicine, Faculty of Dental Medicine and Health, J. J. Strossmayer University of Osijek, 31000 Osijek, Croatia; 4 Department of Periodontology, Faculty of Dental Medicine, University of Rijeka, 51000 Rijeka, Croatia; 5 Department of Pediatric Dentistry, Faculty of Dental Medicine, University of Rijeka, 51000 Rijeka, Croatia; 6 Department of General Pathology and Pathological Anatomy, Faculty of Medicine, University of Rijeka, 51000 Rijeka, Croatia

**Keywords:** late hypersensitivity immune reactions, gingival tissue, gingival enlargement, paraffin-embedded gingival samples, immunohistochemistry, allergic sensitisation, orthodontic appliance, titanium, nickel

## Abstract

This study aimed to analyse the immunohistochemical profile of inflammatory infiltrates in the gingival tissue of patients undergoing orthodontic treatment in relation to patients’ titanium and/or nickel allergy status. Patients with gingival enlargement received initial periodontal therapy, followed by external gingivectomy in the case of persistent gingival enlargement. The sample included 44 patients (22 had metal allergic sensitisation). Histopathological changes were assessed, and an immunohistochemical analysis was performed on formalin-fixed and paraffin-embedded gingival samples using antibodies against CD1a, CD3, CD4, CD8, CD20, CD68, and CD138. Computer-assisted image analysis was performed to evaluate the positive cell count in the gingival tissue. The gingiva of the sensitised patients was characterised by the absence of multifocal inflammatory infiltrates (*p* < 0.05), while pronounced exocytosis and band-like inflammatory infiltrates were more frequently observed in sensitised patients. In addition, there was an increase in Langerhans cells and T-helper lymphocytes and a decrease in naïve T-lymphocytes, cytotoxic T-lymphocytes, macrophages, and plasma cells in the sensitised subjects compared to non-sensitised. However, the differences were only statistically significant for macrophages, with a moderate effect size (82.8 vs. 133.9; *p* = 0.041; r = 0.308). The absence of multifocal inflammation appears to be the most characteristic histopathological feature of the gingiva of sensitised patients. Although their gingiva presented certain characteristics of late hypersensitivity immune reactions the observed changes imply dominant irritative effect e.

## 1. Introduction

During orthodontic treatment, patients often experience gingival enlargement, which could be caused by poor biofilm control and intake of certain medications or as a consequence of the irritating effect of nickel or metal allergy [1,2,3]. Nickel and titanium are the constituent elements of orthodontic alloys. Due to the oral corrosion of alloys during orthodontic treatment, allergic reactions from titanium and/or nickel can occur [4]. The spectrum of signs and symptoms of oral contact allergy is broad, with no specific or pathognomonic clinical picture, making the diagnosis of allergic stomatitis particularly difficult [5]. Allergic contact stomatitis can also occur in the form of a lichenoid contact reaction induced by T-lymphocyte-mediated immune hypersensitivity reactions at sites on the oral mucosa that are in direct contact with the metal in the mouth [6]. The clinical presentation of oral allergic reactions in patients undergoing orthodontic treatment is quite rare and is observed in approximately 0.1–0.2% of patients [7]. Significantly higher doses of nickel are required to elicit reactions in the oral mucosa as compared to the skin [8]. However, titanium has the opposite effect as it penetrates the oral mucosa well, unlike the skin [9]. The mechanisms of delayed-type hypersensitivity reactions are mostly studied in patients with allergic contact dermatitis and allergic reactions to orthopaedic implant metals but not in patients undergoing orthodontic treatment [10,11]. Delayed-type hypersensitivity reactions are mediated by antigen-specific T-lymphocytes. T-helper lymphocytes control cellular immunity by recognising antigens and releasing cytokines to activate effector cells. Although the delayed-type hypersensitivity is mostly associated with T-helper type 1 lymphocytes, other phenotypic T cells, such as cytotoxic lymphocytes, can also be involved [12]. The immunohistochemical and morphological characteristics of the inflammatory infiltrate in the gingiva of patients with metal allergic sensitisation have not been completely understood or investigated. Therefore, this study aimed to assess the histopathological changes and immunohistochemical profile of inflammatory infiltrates in the gingival tissue of patients undergoing orthodontic treatment with confirmed metal allergy and to compare it with those undergoing orthodontic treatment without any metal allergy. The hypothesis was that gingival enlargement could be caused by metal allergy in patients with a confirmed allergy status and that such enlarged gingival tissue could be differentiated from that caused by dental biofilm or irritation. We expected a greater number of T-helper type I lymphocytes, Langerhans cells, and macrophages along with a greater degree of epithelial hyperplasia, exocytosis, and pronounced fibrosis in the gingiva of allergic patients.

## 2. Materials and Methods

### 2.1. Case Selection

A total of 250 patients undergoing orthodontic treatment in three orthodontic offices in Croatia were invited to participate in a study on the prevalence and characteristics of metal allergic sensitisation, out of which 245 accepted the invitation [13]. An external gingivectomy was performed in patients with gingival hyperplasia that was persistent after initial periodontal therapy. Gingival tissue was analysed in 22 patients with confirmed allergic sensitisation to titanium and/or nickel and 22 controls that were not sensitised to titanium and/or nickel (44 patients in total). A minimum sample size of 20 patients per group was needed to detect a difference in the number of antibodies between the two groups and a standard deviation of 20 in each group; therefore, 22 patients were recruited per group. The inclusion criterion was treatment with a fixed orthodontic appliance and persisting gingival enlargement, while the exclusion criteria included the presence of diabetes, endocrine and autoimmune diseases, using medications that affect gingival status (e.g., antiepileptics), and the practice of water sports. All three orthodontic offices used the same type of orthodontic brackets (Ortho Classic, Las Vegas, NV, USA) and nickel titanium wires (GAC International, Islandia, FL, USA). The age range was 11–45 years (median age: 18, interquartile range: 16–22); 68% were female participants, and 52% were adolescents. 

### 2.2. Allergy Testing

The allergy testing included an epicutaneous patch test on nickel sulphate, titanium, titanium dioxide, titanium oxalate, and titanium nitride, with petrolatum as a control (Chemotechnique Diagnostics, Vellinge, Sweden). Testing was performed four months on average after bonding the fixed orthodontic appliance. The upper arm skin was cleaned using medical petrol, and the patch was applied for two days. Skin reactions were evaluated and documented on the second, fourth, and seventh day after applying patch test to confirm an allergic reaction or lack of irritation. If the skin reactions and itching were exacerbated during the evaluation period, this was considered an allergic reaction; in contrast, reactions which lessened over time were regarded as irritations.

### 2.3. Histopathological Analyses

Gingival samples were fixed with 10% neutral buffered formalin, processed by a paraffin-embedding histological technique, and stained with haematoxylin and eosin. Pathohistological changes in the gingiva were analysed, including epithelial and stromal changes, intensity of fibrosis, and intensity and localisation of the inflammatory infiltrate.

### 2.4. Immunohistochemistry

The immunohistochemical envision method was used to analyse the composition of the inflammatory infiltrate using primary antibodies, as listed in Table 1, and an automated immunostainer (Autostainer Link 48+, Agilent Dako, Santa Clara, CA, USA) was used according to the manufacturer’s instructions [14].

Following deparaffinisation in xylene and rehydration in alcohols, 5 μm thick sections were subjected to a heat-induced pre-treatment using PT LINK, according to the manufacturer’s instructions (Agilent Dako, Santa Clara, CA, USA). Envision Flex, high pH (link) K8000 (Agilent Dako, Santa Clara, CA, USA) was used to visualise the primary antibody binding. For each gingival sample and antibody, one negative control slide was prepared by substituting the antibody diluent for the primary antibody (Antibody diluent, S0809, Agilent Dako, Santa Clara, CA, USA).

Immunohistochemical staining was performed using computer-assisted image analysis (CAIA) to evaluate the number of positive cells in the gingival tissue for each immunohistochemical marker (Figures S1–S5). The structures were quantified using an integrated Alphelys Spot Browser 2 system, consisting of an automated microscope (Nikon Eclipse 50i, Nikon Corporation, Tokyo, Japan) with a mounted Microvision CFW-1310C digital camera (Microvision Instruments, Evry, France), 24 bits, 1360 × 1024 pixel resolution under the control of the computer program Alphelys Spot Browser 2.4.4 (Alphelys, Plaisir, France). The system was calibrated using the Nazca program (Microvision instruments, Cedex, France) by determining the point size for each lens (0.3311 µm for 20x lens and 3.320 µm for 2x). After reviewing the entire specimen at 20x magnification, the photographs were taken in selected areas at 100x/200x magnification and were immediately analysed in the program, with constant camera, microscope, and brightness settings.

During the analysis of digital photographs, objects were detected based on colour (wavelength, intensity, and saturation), grouping, and morphological properties (size and shape) marked with colours, which enabled the control and correction of the detection process. For this purpose, several detection algorithms have been developed to define the detection conditions for different objects of interest for quantification. During the detection process, the program automatically measured the detected objects and calculated the default parameters, such as the number, area, and colour intensity.

### 2.5. Statistical Analysis

The χ2 test was used to compare the frequencies of individual histopathological findings. The Mann–Whitney U test was used to compare the intensity of immunohistochemical markers. The effect size was calculated using the Mann–Whitney test according to the formula r = Z/√N. Cramer’s V was used for the effect size in the χ2 and Fisher tests. The following criteria were used in the interpretation: r = 0.1–0.3 = small effect size, 0.3–0.5 = medium, 0.5–0.7 = large, and > 0.7 = very large. All statistical analyses were performed using the IBM SPSS 22 software (IBM Corp., Armonk, NY, USA).

## 3. Results

### 3.1. Pathohistological Characteristics of Gingival Tissue Samples

The gingiva of patients with metal allergic sensitisation had a stronger degree of inflammation, a milder degree of fibrosis, and more frequent epithelial changes (mild hyperplasia and colliquation of the basal layer), exocytosis, and band-like inflammatory infiltrates compared to that of non-sensitised patients. However, most pathohistological changes did not differ significantly between the sensitised and non-sensitised groups.

The gingiva of the sensitised patients more frequently presented with pronounced exocytosis, individual inflammatory cells, band-like inflammatory infiltrates, and colliquation compared to that of non-sensitised patients (Table 2 and Table 3). Multifocal inflammation was less frequently present in the sensitised group than in the non-sensitised group, with a moderate effect size (13.6 vs. 45.5%; *p* = 0.045; V = 0.349). In addition, the gingiva of sensitised patients had less pronounced epithelial hyperplasia, epithelial spongiosis, moderate and severe fibrosis, focal inflammation, multifocal inflammation, inflammation along the basement membrane and deep in the stroma, and mixed inflammatory infiltrate.

### 3.2. Immunohistochemical Features of Gingival Tissue Samples

Generally, regardless of the patient group, the most predominantly observed inflammatory cells in the gingival tissue samples were T-lymphocytes, macrophages, cytotoxic T-lymphocytes, and T-helper lymphocytes. B-lymphocytes were less abundant compared to T-lymphocytes.

**Figure 1:**
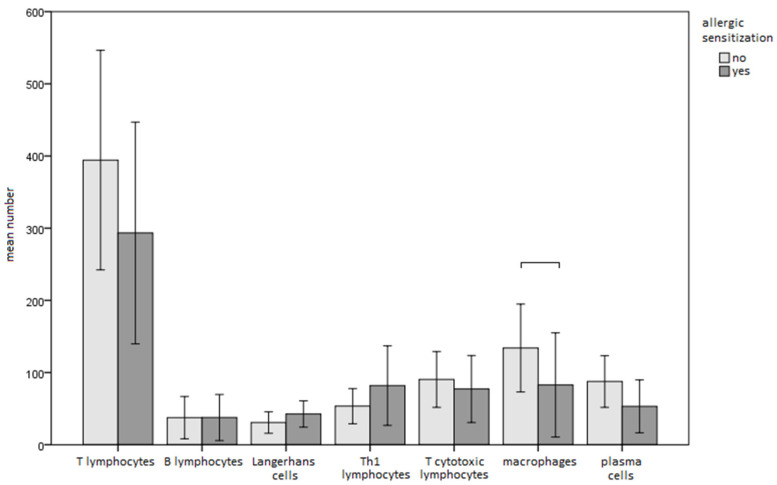
Comparison of immunohistochemical parameters between sensitised and non-sensitised subjects (bars represent means and whiskers 95% confidence intervals).

**Figure 2:**
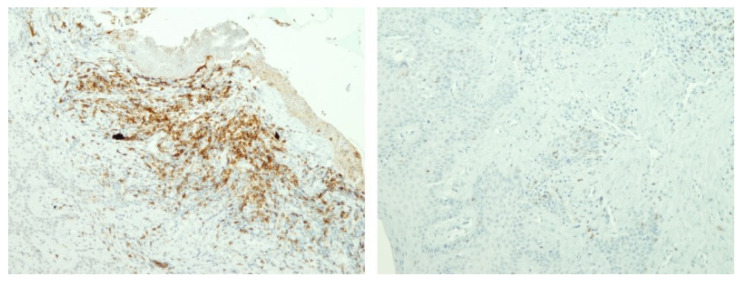
Example samples of immuno-staining for T-helper I lymphocytes in sensitised and non-sensitised patients (Expression of CD4 molecule (T-helper I lymphocytes) is in brown. On the left gingival tissue of sensitised patient, while on the right gingival tissue of non-sensitised patient).

**Figure 3:**
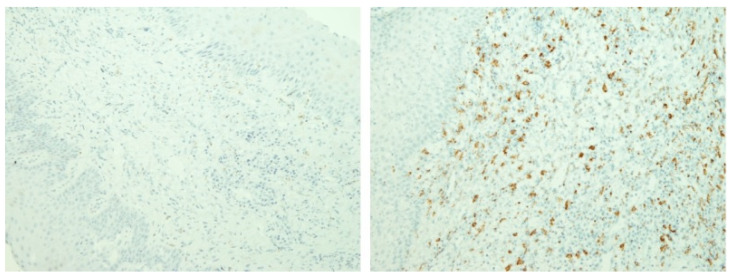
Example samples of immuno-staining for macrophages in sensitised and non-sensitised patients. (Expression of CD68 molecule (macrophages) is in brown. On the left gingival tissue of sensitised patient, while on the right gingival tissue of non-sensitised patient).

There was a tendency towards an increase in Langerhans cells and T-helper I lymphocytes and a decrease in T-lymphocytes, cytotoxic T-lymphocytes, macrophages, and plasma cells in sensitised cells compared to non-sensitised cells. However, the differences were only statistically significant for macrophages, with a moderate effect size (82.8 vs. 133.9; *p* = 0.041; r = 0.308; Figure 1, Figure 2 and Figure 3.) Similar findings were observed when the samples were sub-analysed separately by sex and the type of metal to which the patient was sensitised.

## 4. Discussion

This study showed that the gingiva of patients with metal allergic sensitisation had a higher degree of inflammation, more frequent epithelial changes, and band-like inflammatory infiltrates. However, multifocal inflammation was less often observed in sensitised gingival tissue than in non-sensitised gingival tissue. In addition, the immunohistological findings showed a tendency towards an increase in Langerhans cells and helper T-lymphocytes and a decrease in macrophages, and plasma cells in sensitised cells compared to non-sensitised cells.

Nickel is the most common contact allergen and, like titanium, is a transition metal. Therefore, the tissue changes it causes can be observed uniformly [15,16].

These findings are consistent with the results of a few previous studies that examined nickel allergy in orthodontic patients and reported how the gingiva of sensitised patients responded with inflammation and hyperplasia accompanied by hyperaemia and oedema [17,18]. Studies of cellular, humoral, and histopathological changes in rats using orthodontic appliances revealed increased leukocyte levels alone, mostly due to an increase of monocytes [19]. Hyperplasia also occurs on the stimulation of T-lymphocytes that produce several cytokines, such as IFN-γ, IL-2, IL-5, and IL-10, subsequently leading to cell proliferation [20]. However, the limitation of the abovementioned study was that it studied histopathological characteristics in an animal rat model, which is difficult to generalise in humans.

Histopathological analyses of gingival enlargement in orthodontic patients were also performed in another study, which showed a thinner parakeratinised superficial epithelium and stroma with dense irregularly intertwined collagen and a chronic inflammatory infiltrate. However, the mentioned study did not record whether the patients were sensitised to nickel or titanium [21]. Dental biofilm-induced gingival enlargement shows changes similar to those in the group of orthodontic patients with dense, more regularly distributed subepithelial collagen that limits the zone of chronic inflammatory infiltrates with plasma cells and lymphocytes [21]. The similarities in these groups are quite logical since a good proportion of orthodontic patients present gingival enlargement owing to the biofilm and poor maintenance of oral hygiene. Histologically, the gingiva in orthodontic patients shows a well-structured and thickened epithelium with elongated papillae inserted into the dense fibrous connective tissue [22]. However, the sensitised patients were not differentiated in this study.

The study participants also revealed the presence of epithelial thickening in the form of hyperplasia and connective tissue fibrosis, which was milder in the sensitised group. Previous studies did not define the appearance of the inflammatory infiltrate, and the presence of banded inflammatory infiltrates in sensitised patients may be due to the organisation of inflammatory cells, similar to those observed in the lichenoid reaction. Earlier studies have also reported band-like lichenoid infiltrate in oral contact allergies [23]. The pronounced exocytosis of lymphocytes observed in this study was also reported in another histological study on oral contact allergy [5].

Undoubtedly, nickel is associated with gingival enlargement in orthodontic patients, while titanium represents a new area of research, as it has been considered anti-allergenic until recently. Titanium has been reported to activate macrophages directly or through phagocytosis. Phagocytosis-activated macrophages secrete both pro-inflammatory and anti-inflammatory cytokines, resulting in an imbalance that is implicated in several diseases and allergic reactions [24,25]. It has been shown that titanium particles can increase the expression of certain chemokines in macrophages in vitro [26]. A study examining the tissues of patients with a revision of hip implants found that several titanium particles as well as macrophages, and T-lymphocytes were observed in the absence of B-lymphocytes in hip prosthesis rejection, suggesting titanium sensitisation [10]. However, other studies did not find significant differences in the tissues of titanium-sensitised patients [27]. Nickel can act as an irritant and possibly accumulate in the epithelial and connective tissues of the oral mucosa during orthodontic therapy; gingival enlargement in orthodontic patients, whether metal-sensitive or not, could be primarily caused by the irritating effect of nickel [28]. This could explain the recurrence of gingival enlargement after gingivectomy, which is often observed in non-sensitised patients. In addition, the accumulation of released nickel is enhanced in the dental biofilm of orthodontic patients; therefore, an irritating effect may be present even in the absence of allergic sensitisation. The pathogenetic mechanisms of allergic and irritant contact dermatitis have also been investigated, and it is thought that different initiating events activate similar amplification mechanisms, resulting in almost stereotypical inflammatory pathways involving T-lymphocyte activation independent of an exogenous antigen [29]. This may explain why this study did not report statistically significant differences between the parameters of the sensitised and non-sensitised patients.

Presented immunohistochemical analyses showed that the gingiva of the sensitised patients had a greater number of Langerhans cells and T-helper lymphocytes and fewer naive T-lymphocytes, cytotoxic T-lymphocytes, macrophages, and plasma cells than that of non-sensitised patients. However, the only statistically significant difference was noted due to the decreased macrophage count of the sensitised group.

This study showed an increased number of Langerhans cells in patients sensitised to metals, which corresponds with the fact that Langerhans cells are responsible for antigen presentation. The induction of contact hypersensitivity largely depends on the density of Langerhans cells [30], and an increased number of Langerhans cells in contact hypersensitivity has been reported in previous studies [31]. Connective tissues in contact with nickel were found to have a four times higher number of Langerhans cells [32].

The increased number of T-helper lymphocytes in the metal-sensitised patients observed in this study can be explained by the pathophysiological mechanism of the contact hypersensitivity immune response, in which Langerhans cells present antigens to T-helper lymphocytes, which further trigger the mechanisms of late hypersensitivity by secreting various cytokines and chemokines and generating memory T-helper type 1-lymphocytes [12].

Activated sensitised T-helper lymphocytes, through the secretion of inflammatory cytokines and chemokines, stimulate the plasma extravasation and tissue infiltration of various cells, and as the reaction progresses, the tissue profile stabilises. Therefore, at the peak of the hypersensitivity reaction, 80–90% of cellular infiltrates consist of monocytes and macrophages [12]. Our study results did not reveal a higher number of macrophages in sensitised patients. This phenomenon may have occurred in the time frame when the samples were retrieved since gingival hypertrophy can not be differentiated into the inflammatory or allergic type without initial periodontal therapy. Therefore, it is possible that macrophages or naive T-lymphocytes were not detected in the expected amount in the sensitised patient tissues because the peak of the allergic reaction may have already passed at the time of tissue excision. Furthermore, the reason for the higher number of macrophages in the non-sensitised patients could be their role and innate immunity, which may be stimulated by the irritating effect of metals on the gingiva.

Although cytotoxic CD8+ T lymphocytes may participate in delayed hypersensitivity reactions, they were observed in small proportions in the sensitised patients.

The tendency towards decreased number of plasma cells in sensitised patients is logical because they are associated with antibody secretion; they do not participate in the contact hypersensitivity caused by metals.

The reason for the differences in the results of the earlier histological and immunohistochemical studies discussed in our study and only one statistically significant difference in this study probably lies in the discrepancy between the quality of the tissue samples, laboratory methods of the sample processing, histomorphometry, and the size of the analysed gingival tissue samples, which directly depend on the clinically available biopsy field. One possible reason for the inconsistencies between the study results may be the different time frames in which the samples were taken. It is unlikely that the allergic sensitisation and findings in gingival tissue could be influenced by the type of malocclusion, treatment stage, rate of tooth movement, or age, or that it could significantly influence the findings of this study. A limitation of this study is the lack of a control group of patients treated with nickel-free brackets or aligners, which would have helped to test the hypothesis that nickel accumulates in the epithelial and connective tissue of the oral mucosa during orthodontic therapy. It is possible that gingival enlargement in orthodontic patients, whether metal sensitive or not, is primarily caused by the irritating effect of nickel.

However, the strength of this study is the investigation of the allergy statuses of titanium and/or nickel, which is often overlooked in clinical practice. The investigation was performed on human gingival biopsy tissue models using two groups of patients, sensitised and non-sensitised, and immunoprofiling of the inflammatory events was evaluated in the vicinity of the orthodontic appliance. Clinically, since patients with a metal allergy do not experience an allergic onset of gingival enlargement, it is not always necessary to treat them with nickel-free brackets or aligners. Furthermore, in cases of gingival enlargement and confirmation of metal allergy, it is not always mandatory to debond the appliance before meeting the objectives of orthodontic treatment.

## 5. Conclusions

The absence of multifocal infiltrates in gingival tissue appears to be the most prominent histopathological feature of allergic sensitisation to titanium and nickel in orthodontic patients. The gingiva of sensitised patients revealed few immunohistological characteristics of late hypersensitivity immune reactions; however, the observed changes may imply that the irritative effect induced by nickel and biofilm accumulation might be dominant.

## Figures and Tables

**Table 1:** Characteristics of primary antibodies used for immunohistochemistry.

Antibody	Source	Clone and Manufacturer	Dilution and Incubation	Positive Control
Anti-CD1a	Mouse monoclonal IgG_1_	010, Agilent Dako, Santa Clara, CA, SAD	1:40, 30 min	Langerhans cells
Anti-CD3	Rabbit polyclonal	Agilent Dako, Santa Clara, CA, SAD	1:100, 30 min	T-lymphocytes
Anti-CD4	Rabbit monoclonal IgG	SP35, Cell Marque, Rocklin, CA, SAD	1:75, 60 min	T-helper lymphocytes
Anti-CD8	Mouse monoclonal IgG_1_	C8/144B, Agilent Dako, Santa Clara, CA, SAD	Ready to use, 30 min	T-cytotoxic lymphocytes
Anti-CD20	Mouse monoclonal IgG_2a_	L26, Agilent Dako, Santa Clara, CA, SAD	1:200, 30 min	B-lymphocytes
Anti-CD68	Mouse monoclonal IgG_3_	PG-M1, Agilent Dako, Santa Clara, CA, SAD	1:200, 30 min	Macrophages
Anti-CD138	Mouse monoclonal IgG_1_	MI15, Agilent Dako, Santa Clara, CA, SAD	1:100, 30 min	Plasma cells

**Table 2:** Comparison of epithelial histopathological findings and grouping of inflammatory gingival cells of sensitised when compared to non-sensitised subjects.

		Sensitisation Present (N (% When Compared to Non-Sensitised Subjects))	*p* *
Epithelium without changes	No	22 (53.7%)	
	Yes	0 (0%)	0.233
Mild epithelial hyperplasia	No	10 (40%)	
	Yes	12 (63.2%)	0.223
Pronounced epithelial hyperplasia	No	13 (54.2%)	
	Yes	9 (45%)	0.763
Spongiosis	No	19 (51.4%)	
	Yes	3 (42.9%)	1.000
Colliquation	No	17 (45.9%)	
	Yes	5 (71.4%)	0.412
Mild fibrosis	No	12 (44.4%)	
	Yes	10 (58.8%)	0.537
Moderate fibrosis	No	18 (52.9%)	
	Yes	4 (40%)	0.721
Single inflammatory cells	No	12 (42.9%)	
	Yes	10 (62.5%)	0.347
Focal inflammation	No	18 (52.9%)	
	Yes	4 (40%)	0.721
Multifocal inflammation	No	19 (61.3%)	
	Yes	3 (23.1%)	0.045
Band-like inflammatory infiltrates	No	17 (45.9%)	
	Yes	5 (71.4%)	0.412

* Fisher exact test when comparing to non-sensitised subjects.

**Table 3:** Comparison of histopathological findings of gingival inflammation and inflammatory cells of sensitised when compared to non-sensitised subjects.

		Sensitisation Present (N (% When Compared to Non-Sensitised Subjects))	*p* *
Mild exocytosis	No	21 (53.8%)	
	Yes	1 (20%)	0.345
Pronounced exocytosis	No	18 (45%)	
	Yes	4 (100%)	0.108
Inflammation along the basement membrane	No	14 (56%)	
	Yes	8 (42.1%)	0.543
Inflammation in the upper parts of the stroma	No	1 (50%)	
	Yes	21 (50%)	1.000
Inflammation in the deeper parts of the stroma	No	18 (56.2%)	
	Yes	4 (33.3%)	0.310
Inflammation with predominance of lymphocytes and histiocytes	No	13 (23.1%)	
	Yes	9 (42.9%)	0.547
Mixed inflammatory infiltrate	No	10 (23.1%)	
	Yes	12 (57.1%)	0.547

* Fisher exact test when comparing to non-sensitized subjects.

## Data Availability

The data that support the findings of this study are available from the corresponding author, [M.Z.], upon reasonable request.

## Supplementary Materials

The following supporting information can be downloaded at: https://www.mdpi.com/article/10.3390/ma16010081/s1, Figure S1: Example sample of immuno-staining for T-lymphocytes in non-sensitised patient (Expression of CD3 molecule (T-lymphocytes) is in brown.; Figure S2: Example sample of immuno-staining for Langerhans cells in sensitised patient (Expression of CDa1 molecule (Langerhans cells) is in brown.; Figure S3: Example sample of immuno-staining for T-cytotoxic lymphocytes in sensitised and non-sensitised patient (Expression of CD8 molecule (T-cytotoxic lymphocytes) is in brown.; Figure S4: Example sample of immuno-staining for B-lymphocytes in non-sensitised patient (Expression of CD20 molecule (B-lymphocytes) is in brown.; Figure S5: Example sample of immuno-staining for plasma cells in non-sensitised patient (Expression of CD138 molecule (plasma cells) is in brown.
