# Supplementary material for: Immunohistochemical and Histopathological Features of Persistent Gingival Enlargement in Relation to Metal Allergic Sensitisation during Orthodontic Treatment
Source: Materials (Basel). 2022 Dec 22;16(1):81. doi: 10.3390/ma16010081 (PMC9821443; doi:10.3390/ma16010081)
Supplement: Supplementary file 1 [file materials-16-00081-s001.zip › materials-2041243-supplementary.pdf]

materials

Article

Immunohistochemical and Histopathological Features of Persistent Gingival Enlargement in Relation to Metal Allergic Sensitisation during Orthodontic Treatment

Martina Zigante [1,2,*], Stjepan Spalj [1,2,3], Jelena Prpic [2,4], Andrej Pavlic [1,5], Visnja Katic [1,2] and Koviljka Matusan Ilijas [2,6]

[1] Department of Orthodontics, Faculty of Dental Medicine, University of Rijeka, 51000 Rijeka, Croatia
[2] Clinical Hospital Center Rijeka, 51000 Rijeka, Croatia
[3] Department of Dental Medicine, Faculty of Dental Medicine and Health, J. J. Strossmayer University of Osijek, 31000 Osijek, Croatia
[4] Department of Periodontology, Faculty of Dental Medicine, University of Rijeka, 51000 Rijeka, Croatia
[5] Department of Pediatric Dentistry, Faculty of Dental Medicine, University of Rijeka, 51000 Rijeka, Croatia
[6] Department of General Pathology and Pathological Anatomy, Faculty of Medicine, University of Rijeka, 51000 Rijeka, Croatia
* Correspondence: martina.zigante@fdmri.uniri.hr

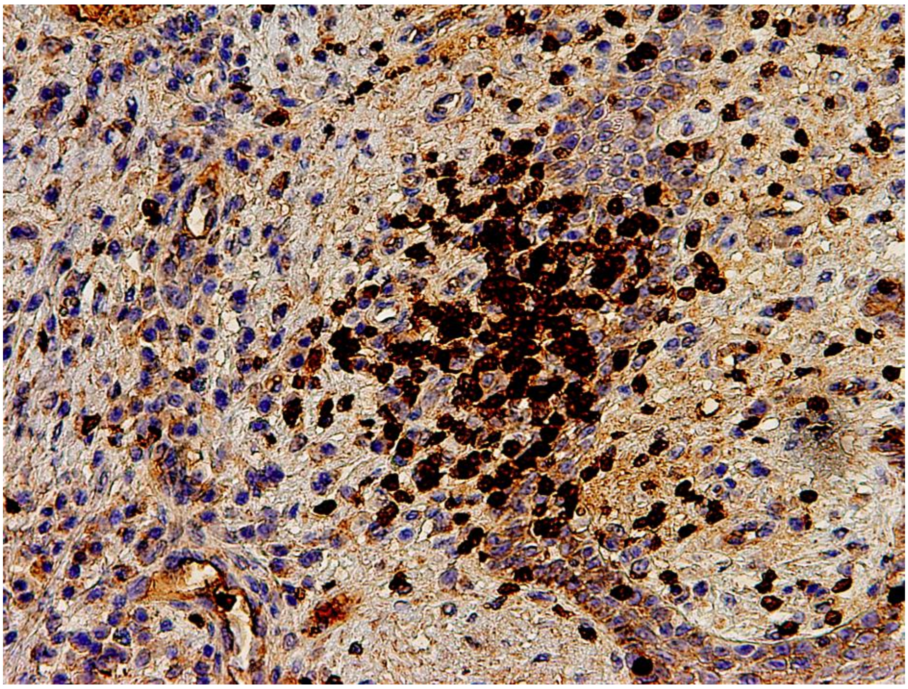

Figure S1. Example sample of immuno-staining for T-lymphocytes in non-sensitised patient (Expression of CD3 molecule (T-lymphocytes) is in brown.

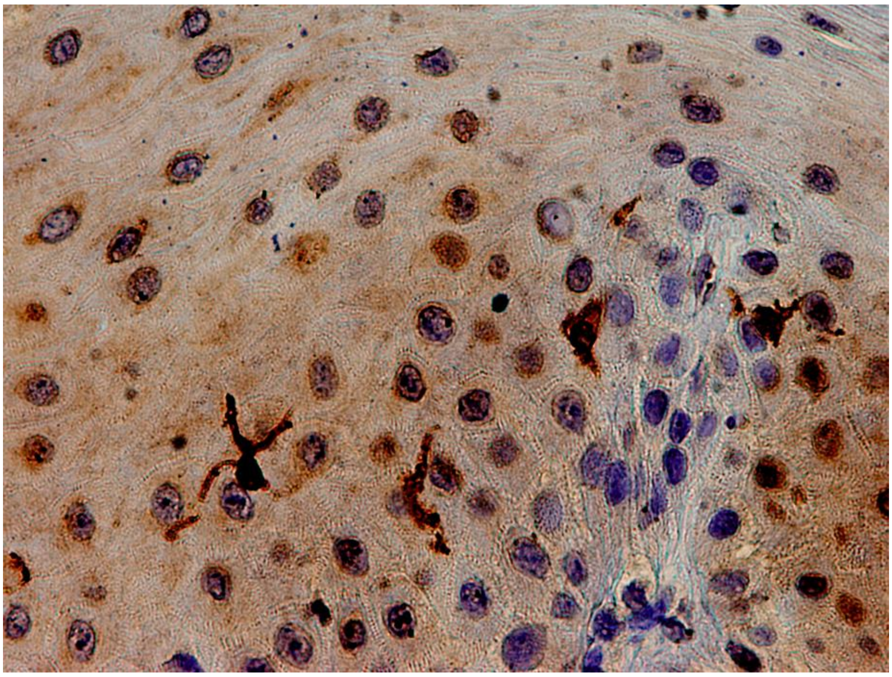

Figure S2. Example sample of immuno-staining for Langerhans cells in sensitised patient (Expression of CDa1 molecule (Langerhans cells) is in brown.

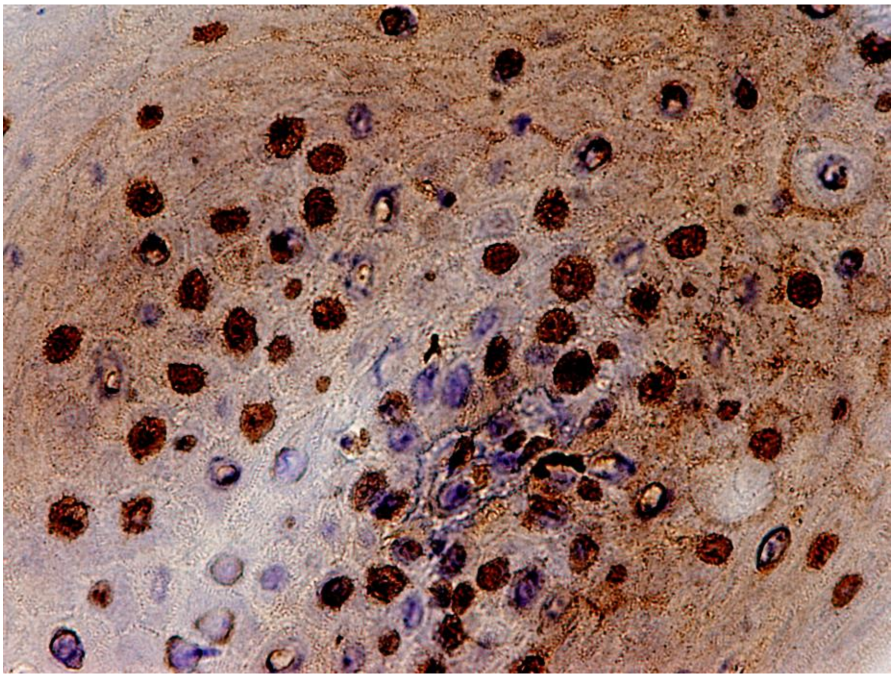

Figure S3. Example sample of immuno-staining for T-cytotoxic lymphocytes in sensitised and non-sensitised patient (Expression of CD8 molecule (T-cytotoxic lymphocytes) is in brown.

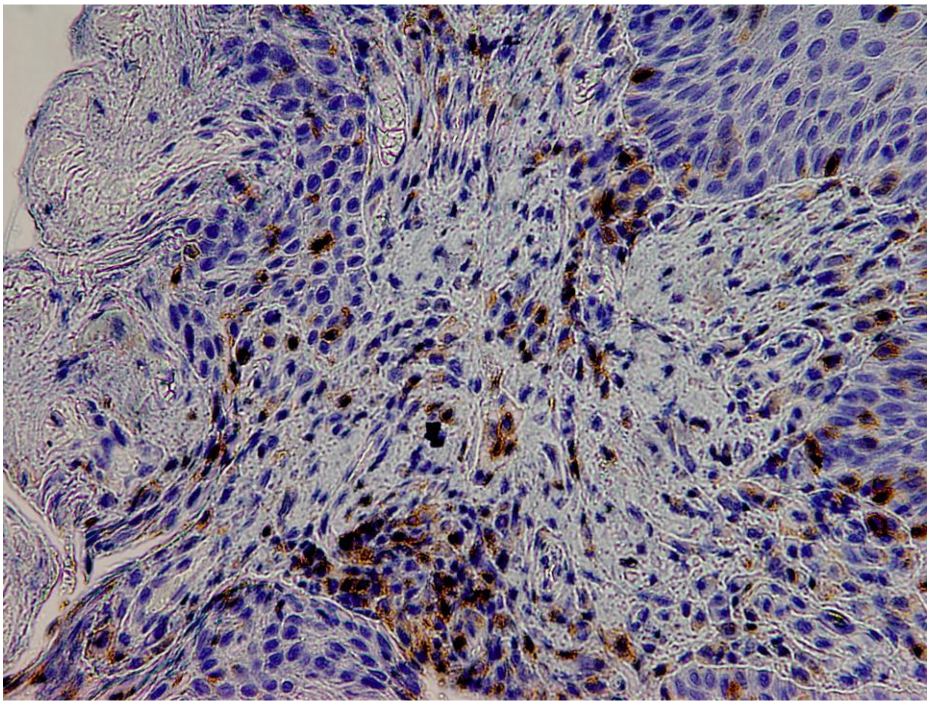

Figure S4. Example sample of immuno-staining for B-lymphocytes in non-sensitised patient (Expression of CD20 molecule (B-lymphocytes) is in brown.

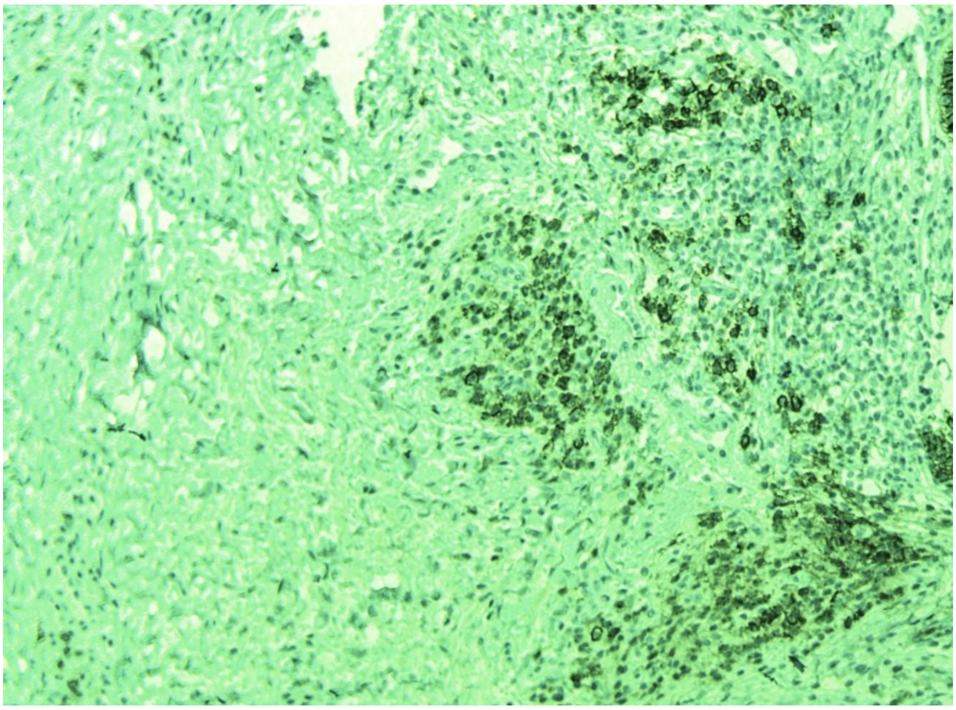

Figure S5. Example sample of immuno-staining for plasma cells in non-sensitised patient (Expression of CD138 molecule (plasma cells) is in brown.
